# The Senolytic Drug Navitoclax Protects the Brain After Experimental Ischemic Stroke

**DOI:** 10.3390/ph19030431

**Published:** 2026-03-06

**Authors:** Dianoush Falahatgaroshibi, Júlia Baixauli-Martín, María C. Burguete, Mikahela A. López-Morales, Alicia Aliena-Valero, José E. Peris, Juan B. Salom

**Affiliations:** 1 Unidad Mixta de Investigación Cerebrovascular, Instituto de Investigación Sanitaria La Fe, 46026 Valencia, Spain; dianosh.felahatgar11@gmail.com (D.F.); juliabaixauli@gmail.com (J.B.-M.); m.consuelo.burguete@uv.es (M.C.B.); salom_jba@gva.es (J.B.S.); 2 Departamento de Biotecnología, Universidad Politécnica de Valencia, 46022 Valencia, Spain; 3 Departamento de Fisiología, Universidad de Valencia, 46100 Burjassot, Spain; 4 Departamento de Fisioterapia, Universidad de Valencia, 46010 Valencia, Spain; 5 Departamento de Farmacia y Tecnología Farmacéutica y Parasitología, Universidad de Valencia, 46100 Burjassot, Spain; jose.e.peris@uv.es

**Keywords:** ischemic stroke, cell senescence, navitoclax, senolysis, cerebroprotection

## Abstract

**Background/Objectives**: Senescence has been recently described in brain cells following ischemic stroke. The potential of targeting senescence as an effective therapeutic approach in the treatment of ischemic stroke requires further investigation. This study evaluated the effects of the senolytic drug navitoclax after experimental ischemic stroke. **Methods**: Navitoclax was injected into male young Wistar rats at doses of 10 and 30 mg/kg (i.p.). to evaluate its pharmacokinetics, cerebral levels and potential to cause thrombocytopenia. Subsequently, a second group of rats underwent 60 min of transient middle cerebral artery occlusion (tMCAO). Navitoclax (10 mg/kg, i.p.) or vehicle was injected every other day between days 3 and 13 after tMCAO. Neurofunctional performance, infarct size, and senescence markers were assessed on day 14. **Results**: Navitoclax (10 mg/kg) administration resulted in a maximum plasma concentration of 0.702 mg/L and half-life of 11.33 h. Additionally, a brain concentration of 0.04 ± 0.02 µg/g was detected. Moderate thrombocytopenia was induced by 10 mg/kg, and to a greater extent by 30 mg/kg. Navitoclax (6 × 10 mg/kg) improved neurofunctional impairment, as indicated by significant decrease by 66% in the total time for the tape removal test, and significantly reduced infarct area by 52% when compared to vehicle. Moreover, navitoclax significantly reduced levels of SA-β-gal (by 80%), lipofuscin (by 91%), and Checkpoint kinase 2 (Chk2; by 69%) in the ischemic hemisphere. **Conclusions**: Navitoclax protects the brain after ischemic stroke by improving neurofunctional outcome and reducing infarct size, which is associated with reducing senescence markers. Although moderate thrombocytopenia warrants caution, targeting senescence emerges as a promising therapeutic strategy for ischemic stroke.

## 1. Introduction

Stroke is the second leading cause of death worldwide, and the third leading cause of death and disability combined, with ischemic stroke being the most prevalent type [1]. The majority of approved therapeutic interventions for diseases are developed to target their underlying pathophysiology. For over three decades, researchers have made tremendous progress in unraveling the molecular and cellular changes that occur after a stroke. Although significant advancements have been made in the treatment of ischemic stroke, including thrombolysis and mechanical thrombectomy, limitations and unmet needs remain. Additionally, some therapies that show promise in preclinical studies often fail to translate effectively to clinical trials. There is unexplored potential in stroke pathogenesis that could be exploited to discover effective cerebroprotective agents through de novo drug development and drug repurposing [2].

Cellular senescence is characterized by stable cell cycle arrest and a hypersecretory state occurring in multiple physiological and pathological processes [3]. Senescence can be induced via intrinsic and extrinsic stimuli, through replicative and stress-induced premature senescence (SIPS), respectively. SIPS occurs in many organs and tissues after ischemia/reperfusion injury [4]. Cellular senescence and senescence-associated secretory phenotype (SASP) are involved in brain injury and neurological degeneration [5]. Ischemic stroke injury causes oxidative stress and mitochondrial damage leading to SIPS in brain cells. Therefore, senescence and its hypersecretory phenotype are potential targets for ischemic stroke treatment [6].

Therapies that target senescent cells, known as senotherapeutics, have emerged as a promising treatment strategy for the central nervous system (CNS). These therapeutic approaches involve removing senescent cells by disabling their pro-survival pathways with senolytics; or suppressing their toxic SASP using senomorphics. As for senolytics, they can deactivate different survival pathways, referred to as senescent cell anti-apoptotic pathways (SCAPs), thus triggering apoptosis [7]. Senolytic navitoclax (ABT-263), is a potent and orally bioavailable BH3 mimetic that functions as an inhibitor of the Bcl-2 family of anti-apoptotic proteins [8]. It exerts its effect by binding to Bcl-2 and Bcl-xL. This facilitates the release of pro-apoptotic proteins, Bax and Bak, thus inducing cell apoptosis. Navitoclax has been shown to reduce biomarkers of senescence and neurodegeneration in aged nonhuman primates. Overall, in this study, navitoclax treatment was safe and well tolerated, though it induced transient moderate thrombocytopenia, consistent with its mechanism of action [9].

We and others have identified brain cell senescence and SASP as new therapeutic targets in rodent models of ischemic stroke [10,11]. Furthermore, we have recently reported on the spatial and temporal characterization of cellular senescence features in experimental ischemic stroke [12]. Evidence for beneficial effects of senolysis with navitoclax in ischemic brain injury is limited but promising [13,14]. Building upon our previous characterization of cell senescence, the present study aimed to investigate the cerebroprotective effects of navitoclax in the rat filament model of transient middle cerebral artery occlusion (tMCAO). This is a rodent model of the thrombectomy technique, the standard endovascular procedure used to treat acute ischemic stroke caused by large vessel occlusion [15]. To this end, we first evaluated in healthy animals: (1) the pharmacokinetics and cerebral levels of navitoclax, and (2) its effect on blood platelet counts. Then, we assessed the effects of navitoclax in animals subjected to ischemic stroke by measuring: (1) neurofunctional performance, (2) infarct size, (3) previously identified markers of cell senescence and SASP, and (4) blood platelet counts.

## 2. Results

### 2.1. Circulating Navitoclax Increased and Reached the Brain After Intraperitoneal Injection

**Figure 1:**
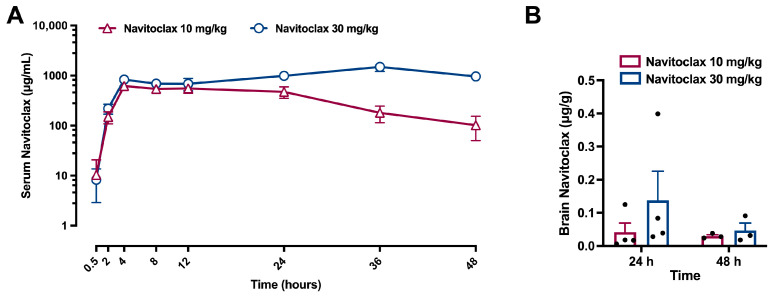
Navitoclax pharmacokinetics and brain levels after injection (10 or 30 mg/kg, i.p.) to naïve adult male Wistar rats. (**A**) Time course of serum navitoclax levels in blood samples collected at 0.5, 1, 2, 4, 8, 12, 24, 36, and 48 h after administration (10 mg/kg, *n* = 5; 30 mg/kg, *n* = 7). (**B**) Navitoclax levels in brain homogenates at 24 or 48 h after administration. Two-way ANOVA followed by Šidák test. Data are expressed as mean ± SEM.

Serum navitoclax levels increased following an i.p. injection of 10 mg/kg (t_max_ = 15 ± 9 h; C_max_ = 0.702 ± 0.169 mg/L; t_1/2_ = 11.3 ± 3.1 h) and to a higher extent after 30 mg/kg i.p. injection (t_max_ = 25 ± 15 h; C_max_ = 1.205 ± 0.431 mg/L; t_1/2_ > 48 h). Figure 1A shows circulating navitoclax levels up to 48 h after administration. In addition, navitoclax was detected in brain tissue 24 and 48 h following both 10 and 30 mg/kg i.p. injections (Figure 1B).

### 2.2. Navitoclax-Induced Thrombocytopenia

**Figure 2:**
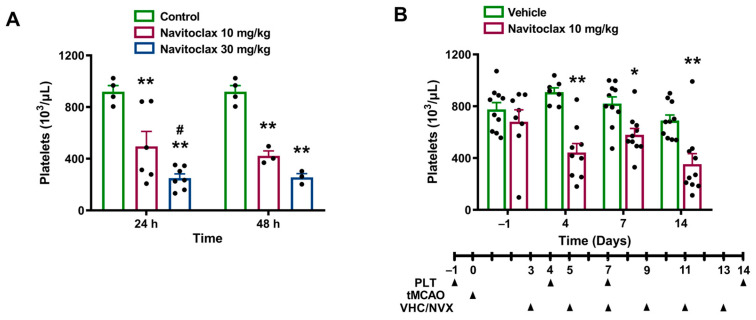
Effects of navitoclax on platelet counts. (**A**) Platelet counts in blood samples collected at 24 and 48 h after navitoclax injection (10 or 30 mg/kg, i.p.) in naïve adult male Wistar rats. Mixed-effects model was used, followed by Šidák test. ** *p* < 0.01, significantly different from control. # *p* < 0.05, significantly different from navitoclax 10 mg/kg. (**B**) Platelet counts in blood samples collected one day before and 4, 7, and 14 days after transient middle cerebral artery occlusion (tMCAO) in adult male Wistar rats. The animals were injected i.p. with navitoclax (10 mg/kg) or vehicle every other day between days 3 and 13 after tMCAO. Mixed-effects model was used, followed by Šidák test. * *p* < 0.05, ** *p* < 0.01, significantly different from vehicle. Data are expressed as mean ± SEM.

An i.p. injection of 10 mg/kg of navitoclax induced a significant reduction in platelet counts at 24 h (54% of control, *p* < 0.01) and at 48 h (45% of control, *p* < 0.01). This effect was significantly more pronounced following the 30 mg/kg i.p. dose, which reduced platelet counts to 26% of control at 24 h (*p* < 0.01) and to 28% of control at 48 h (*p* < 0.01). Figure 2A shows the time course of platelet counts following the administration of a single dose of 10 or 30 mg/kg navitoclax.

Given the pharmacokinetics of navitoclax and its effects on platelet counts, the low dose of 10 mg/kg was chosen for repeated administration after ischemic stroke.

### 2.3. Navitoclax Accelerated Neurofunctional Recovery After Ischemic Stroke

Table 1 shows the cortical perfusion (CP), blood glucose levels, and body weight of the two experimental groups of rats subjected to tMCAO, before i.p. treatment with navitoclax or vehicle. The reduction in CP during ischemia and the extent of perfusion increase during reperfusion was comparable in both groups. Glycemia was in the physiological range and similar in both groups.

**Figure 3:**
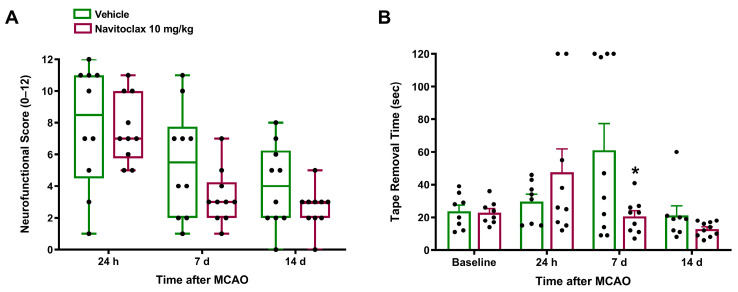
Effects of navitoclax on neurofunctional impairment in stroke adult male Wistar rats 24 h, 7 and 14 days after tMCAO. The animals were injected i.p. with navitoclax (10 mg/kg) or vehicle every other day between days 3 and 13 after tMCAO. (**A**) Neurofunctional score (0–12). Kruskal–Wallis followed by Dunn’s multiple comparisons test. Data are expressed as median (Q1, Q3). (**B**) Total time for tape removal in the bilateral asymmetry test. Mixed-effects model was used, followed by Šidák test. * *p* < 0.05, significantly different from vehicle. Data are expressed as mean ± SEM.

Ischemic stroke induced neurofunctional impairment. Animals subjected to tMCAO exhibited a neurofunctional score of 8.5 (4.5, 11) after 24 h, and their total time in the tape removal test increased 2.57-fold after seven days. Vehicle-treated animals showed spontaneous recovery of neurofunctional impairment in terms of both neurofunctional score and tape removal performance. Six i.p. navitoclax injections (10 mg/kg), administered every other day between days 3 and 13 after tMCAO, resulted in a tendency toward higher improvement in the neurofunctional score at 7 and 14 days post-stroke compared to vehicle (Figure 3A). Furthermore, navitoclax treatment significantly reduced tape removal time at 7 days post-stroke compared to vehicle (*p* < 0.05; Figure 3B). Supplementary Figure S1A shows the reaction time in the tape removal test, which was also significantly reduced at 7 days post-stroke compared to vehicle (*p* < 0.05). As expected, the left forepaw tape removal time, which corresponds to the affected side of the animal, was also increased after 7 days of stroke (Figure S1B), while right forepaw time remained unchanged (Figure S1C). The beneficial effects of navitoclax involved a significant reduction in left forepaw tape removal time (*p* < 0.05; Figure S1B).

### 2.4. Navitoclax Reduced Infarct Size After Ischemic Stroke, Despite Thrombocytopenia

**Figure 4:**
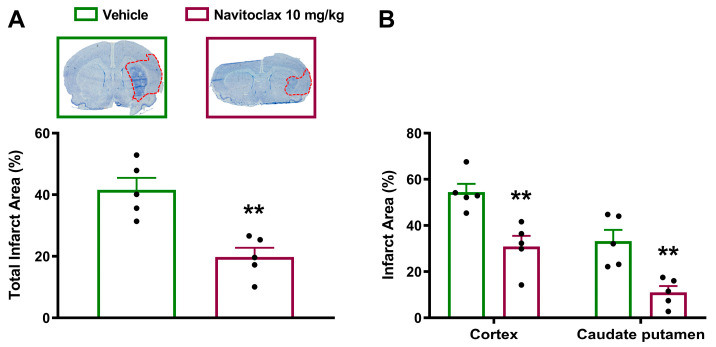
Effects of navitoclax on brain infarct size in adult male Wistar rats 14 days after tMCAO. The animals were injected i.p. with navitoclax (10 mg/kg) or vehicle every other day between days 3 and 13 after tMCAO. (**A**) Representative macroscopic images (2× magnification) of thionine-stained brain coronal sections (0.2 to −1.8 mm from the bregma) in vehicle- and navitoclax-treated animals, and quantification of total infarct area (red dotted lines). Two-tailed Student’s *t*-test. ** *p* < 0.01, significantly different from vehicle. (**B**) Regional quantification of infarct area in the cortex and caudate putamen. Two-way ANOVA followed by Šidák test. ** *p* < 0.01, significantly different from vehicle. Data are expressed as mean ± SEM.

Fourteen days after the ischemic stroke, navitoclax-treated animals showed significantly smaller infarcts compared to vehicle (*p* < 0.01; Figure 4A and Figure S2). Significant reductions in infarct size in both cortex (*p* < 0.01) and caudate putamen (*p* < 0.01) regions accounted for total infarct reduction (Figure 4B). Cerebroprotective effects of navitoclax were found despite a significant reduction in platelet counts observed at days 4 (*p* < 0.01), 7 (*p* < 0.05), and 14 (*p* < 0.01) during repeated administration (Figure 2B).

### 2.5. Navitoclax Reduced Senescence-Associated β-Galactosidase Expression and Lipofuscin Accumulation in the Ischemic Brain

**Figure 5:**
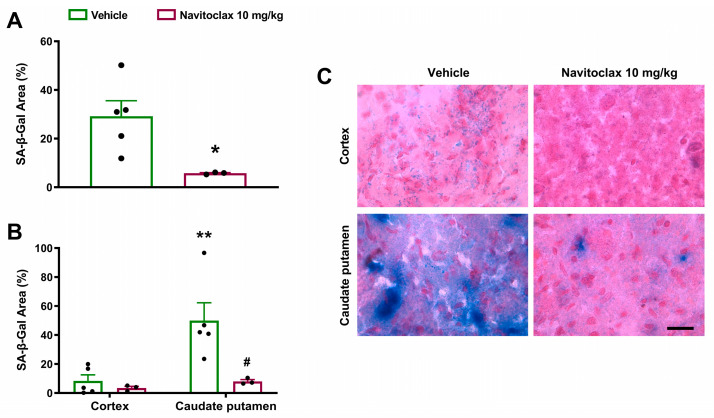
Effects of navitoclax on senescence-associated β-galactosidase (SA-β-gal) expression in the brain of adult male Wistar rats 14 days after tMCAO. The animals were injected i.p. with navitoclax (10 mg/kg) or vehicle every other day between days 3 and 13 after tMCAO. (**A**) SA-β-gal expression area in coronal sections (0.2 to −1.8 mm from the bregma) of vehicle- and navitoclax-treated animals. Two-tailed Student’s *t*-test. * *p* < 0.05, significantly different from vehicle. (**B**) Regional quantification of SA-β-gal expression area in the cortex and caudate putamen. Two-way ANOVA followed by Tukey’s test. ** *p* < 0.01, significantly different from cortex. # *p* < 0.05, significantly different from vehicle. Data are expressed as mean ± SEM. (**C**) Representative images of the microscopic fields in the SA-β-gal-stained cortex and caudate putamen of vehicle- and navitoclax-treated animals. Scale bar = 10 μm.

Fourteen days after the ischemic stroke, senescence-associated β-galactosidase (SA-β-gal) expression was observed in the ischemic hemisphere (Figure 5A). Expression was significantly higher in the caudate putamen than in the cortex (*p* < 0.01; Figure 5B,C). Repeated navitoclax treatment significantly reduced SA-β-gal expression in the ischemic hemisphere (*p* < 0.05; Figure 5A) due primarily to a significant reduction in SA-β-gal expression in the caudate putamen (*p* < 0.05; Figure 5B,C).

**Figure 6:**
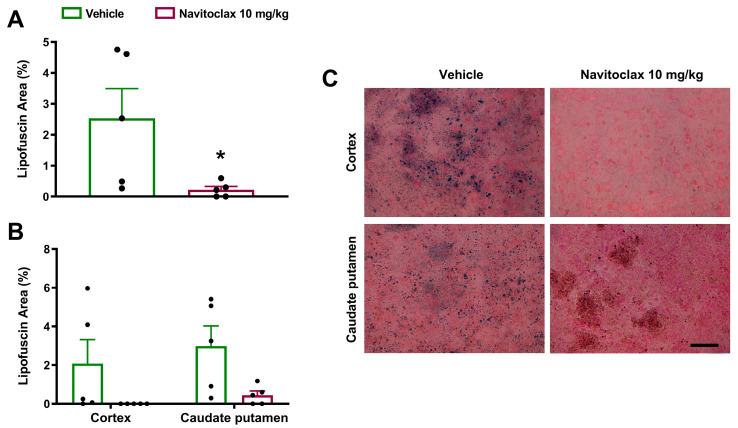
Effects of navitoclax on lipofuscin accumulation in the brain of adult male Wistar rats 14 days after tMCAO. The animals were injected i.p. with navitoclax (10 mg/kg) or vehicle every other day between days 3 and 13 after tMCAO. (**A**) Lipofuscin accumulation area in coronal sections (0.2 to −1.8 mm from the bregma) of vehicle- and navitoclax-treated animals. Two-tailed Student’s *t*-test. * *p* < 0.05, significantly different from vehicle. (**B**) Regional quantification of lipofuscin accumulation area in the cortex and caudate putamen. Two-way ANOVA followed by Tukey’s test. Data are expressed as mean ± SEM. (**C**) Representative images of the microscopic fields in the Sudan Black B-stained cortex and caudate putamen of vehicle- and navitoclax-treated animals. Scale bar = 25 μm.

There was highly variable accumulation of lipofuscin granules in the ischemic hemisphere fourteen days after the ischemic stroke (Figure 6A). Lipofuscin granules occurred to a similar extent in the cortex and caudate putamen (Figure 6B,C). Repeated navitoclax treatment significantly reduced lipofuscin accumulation in the ischemic hemisphere (*p* < 0.05; Figure 6A) by reducing lipofuscin granules in the cortex and caudate putamen (Figure 6B,C). Similar results were obtained by measuring the number of lipofuscin particles, instead of total lipofuscin area (Figure S3).

### 2.6. Navitoclax Reduced Checkpoint Kinase 2 Expression in the Ischemic Brain

**Figure 7:**
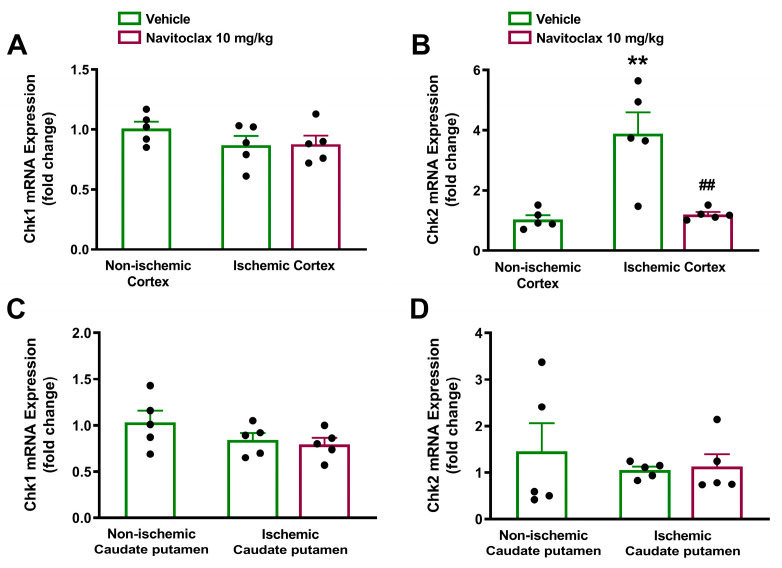
Effects of navitoclax on the mRNA expression of checkpoint kinases (Chk1 and Chk2) in the brain of adult male Wistar rats 14 days after tMCAO. The animals were injected i.p. with navitoclax (10 mg/kg) or vehicle every other day between days 3 and 13 after tMCAO. (**A**) Chk1 expression in non-ischemic and ischemic cortex of vehicle- and navitoclax-treated animals. (**B**) Chk2 expression in non-ischemic and ischemic cortex of vehicle- and navitoclax-treated animals. (**C**) Chk1 expression in non-ischemic and ischemic caudate putamen of vehicle- and navitoclax-treated animals. (**D**) Chk2 expression in non-ischemic and ischemic caudate putamen of vehicle- and navitoclax-treated animals. One-way ANOVA followed by Tukey’s test. ** *p* < 0.01, significantly different from non-ischemic cortex. ## *p* < 0.01, significantly different from vehicle. Data are expressed as mean ± SEM.

The expression of cell senescence and SASP biomarkers at mRNA level was measured in the ischemic hemisphere 14 days after stroke. At this time point, the cell cycle regulator Chk2, but not Chk1, was significantly overexpressed in the ischemic compared to non-ischemic cortex (*p* < 0.01; Figure 7A,B). Expression of both checkpoint kinases remained unchanged in the caudate putamen (Figure 7C,D). Repeated navitoclax treatment significantly inhibited Chk2 upregulation in the ischemic cortex (*p* < 0.01; Figure 7B).

Fourteen days after the ischemic stroke, the mRNA expression levels of the cyclin-dependent kinase inhibitors p16 and p21 (Figure S4) and the SASP cytokines IL-1β and TNF-α (Figure S5) were not significantly different between the ischemic and non-ischemic hemispheres, in either the cortex or the caudate putamen. Repeated navitoclax treatment had no effect on the expression of these senescence and SASP biomarkers.

## 3. Discussion

The present study shows the cerebroprotective effects of intermittent navitoclax senolytic treatment after transient middle cerebral artery occlusion in a rat model that mimics the endovascular procedure of mechanical thrombectomy in patients with large vessel occlusion ischemic stroke. Navitoclax, a small-molecule inhibitor of the Bcl-2 family of anti-apoptotic proteins [8], accelerated neurofunctional recovery and reduced infarct size fourteen days after ischemic stroke. These cerebroprotective effects were associated with a reduction in SA-β-gal expression, in lipofuscin accumulation, and in Chk2 expression in the ischemic brain.

Our present results showing navitoclax’s ability to reduce brain infarct size measured two weeks after ischemic stroke are in line with previous findings showing smaller infarct size four days post-stroke [14]. This supports a sustained effect of navitoclax on brain damage. Regarding the neurofunctional benefits of navitoclax, our results using a 12-point neurofunctional scale align with previous findings using an 18-point scale in rats [14] and mice [13]. Similarly, better performance in the tape removal test also agrees with prior findings in rats using the limb placement test, which also evaluates sensorimotor performance [14]. Moreover, the beneficial effects of navitoclax have also been reported in mice using the rotarod and the locomotor activity tests [13].

We previously reported two major biomarkers of cell senescence, SA-β-gal expression and lipofuscin accumulation, in the ischemic brains of rats [10,12]. In the present study, we discovered that reductions in brain levels of SA-β-gal and lipofuscin, the two most common and recognizable cell senescence markers for dysfunctional lysosomes [16], were associated with navitoclax’s cerebroprotective effects. In addition, we previously found increased genic expression of established markers of senescence at earlier time points after ischemic stroke [10,12]. In the present study, two weeks after the ischemic insult, only the expression of Chk2 was found increased at the transcriptional level, as expected from prior results. It is possible that changes in the expression of cell cycle markers, such as p16, could have been captured at earlier time points than 14 days. Checkpoint kinases can be part of the DNA segments with chromatin alterations reinforcing senescence (DNA-SCARS) implicated in both senescence-mediated cell cycle arrest and SASP [17,18]. The increase in the Chk2 gene expression could be attributed to the persistence of the DNA-SCARS in the senescent cells. Alternatively, and based on our previous spatio-temporal study [12], we hypothesize that the observed Chk2 increase could be the reflection of cell populations that have undergone a process of secondary senescence over time (e.g., microglia). Navitoclax treatment also reduced the late Chk2 expression in the ischemic brain. This may be a consequence of the elimination of primary senescent cells induced by navitoclax treatment, which prevents senescence from spreading to neighboring cells.

Navitoclax has been usually administered as one or more cycles of repeated doses in rodent models of tumor xenograft [8], aging [19], and age-related neurodegenerative disease [20], although single doses have also been used in traumatic brain injury [21]. To select a dosing regimen for navitoclax, we first assessed its pharmacokinetics and potential thrombocytopenic effects following intraperitoneal single-dose administration. After 10 mg/kg injection, circulating navitoclax increased to 0.702 mg/L, with a half-life of almost 12 h, and detectable serum levels up to 48 h after injection. We therefore chose a schedule for navitoclax treatment consisting of six i.p. injections of 10 mg/kg every other day between days 3 and 13 after ischemic stroke. This regimen is quite similar to those previously used in rodent models of ischemic stroke [13,14] and myocardial infarction [22].

Navitoclax was detected in the rat brain 24 and 48 h following intraperitoneal administration, thus showing that it has sufficient blood–brain barrier (BBB) permeability to have direct CNS effects. Our results further support previous findings regarding navitoclax’s brain penetration following oral gavage administration in healthy rats and mice [9]. In our study, navitoclax was injected starting 3 days after stroke onset, when BBB permeability was facilitated by ischemia-induced BBB breakdown [23].

In the present study, a single dose of navitoclax injected into healthy rats induced dose-dependent, moderate thrombocytopenia. It is noteworthy that thrombocytopenia was not exacerbated in animals that received the six-dose intermittent treatment regimen following ischemic stroke. Navitoclax-induced thrombocytopenia is consistent with its mechanism of action by inhibiting Bcl-2 family proteins, which are essential for platelet survival [24]. Similar reductions in circulating platelet counts have been reported in dogs [8] and aged cynomolgus monkeys [9] following navitoclax administration, with recovery to normal levels after treatment cessation. Despite thrombocytopenia, navitoclax reduced infarct size and neurofunctional impairment after ischemic stroke; the galacto-conjugation of navitoclax as a strategy to increase senolytic specificity and reduce platelet toxicity [25] deserves further research.

Apart from inhibiting Bcl-2 family proteins, other pro-survival/anti-apoptotic pathways can be targeted to induce senolysis. The combination of dasatinib (Src tyrosine kinase inhibitor) with quercetin (PI3K inhibitor) synergistically influenced SCAPs and demonstrated significant senolytic potential [7]. It has been recently reported that senolytic treatment with dasatinib plus quercetin attenuates brain injury and enhances cognitive recovery in a mouse model of global ischemia [26]. Navitoclax was selected for use in this study as it is one of the most studied senolytic drugs. Future research could address the potential cerebroprotective effect of dasatinib plus quercetin treatment, alone or in combination with navitoclax, in rodent models of ischemic stroke.

Finally, a limitation of this study is that the experiments were conducted exclusively with male rats. Further studies involving experiments with female rats are needed to assess whether brain senescence following ischemic stroke exhibits sexual dimorphism that could influence the response to senolytic treatment with navitoclax.

In conclusion, senolytic treatment with navitoclax protects the brain after ischemic stroke by reducing infarct size and improving neurofunctional outcome. This is consistent with a reduction in common and recognizable markers of cell senescence in the ischemic brain. Although moderate thrombocytopenia warrants caution, targeting senescence emerges as a promising cerebroprotective therapeutic strategy for ischemic stroke.

## 4. Materials and Methods

### 4.1. Animals and Ethical Issues

**Figure 8:**
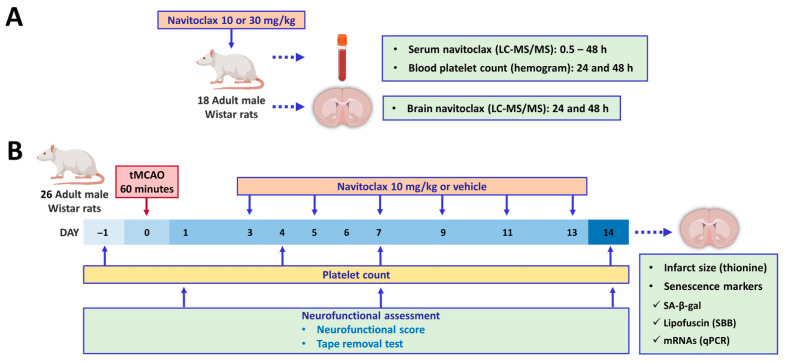
Outline of experimental paradigm. (**A**) Naïve adult male Wistar rats were used to assess the pharmacokinetics of navitoclax, its ability to reach brain tissue, and its potential to induce thrombocytopenia. (**B**) Adult male Wistar rats underwent tMCAO and were randomized to receive either vehicle or navitoclax injections. Neurofunctional evaluations and platelet counts were performed prior to euthanasia. Then, brains were collected to assess infarct size and cell senescence markers.

A total of 44 male 12-week-old Wistar rats from Charles River (Barcelona, Spain) were used in the study (Figure 8). They were housed under standard conditions, including ad libitum feeding and drinking, and 12 h light/12 h dark cycle (light was turned on at 8:00 h and turned off at 20:00 h). The study was designed and conducted according to the STAIR/RIGOR guidelines [27,28] regarding physiological monitoring, simple randomization, predefined exclusion criteria, allocation concealment, blinded assessment of several outcomes at different endpoints, and conflict of interest statement. The ARRIVE (Animal Research: Reporting of In Vivo Experiments) guidelines were followed (https://arriveguidelines.org/) (accessed on 1 March 2026). Experiments were conducted in compliance with the legislation on protection of animals used for scientific purposes in Spain (RD 53/2013) and the EU (Directive 2010/63/EU). Protocols were approved by the Animal Experimentation Ethics Committee from IIS La Fe (2020-363-1).

### 4.2. Experimental Groups

Figure 8 shows the experimental paradigm. Eighteen naïve animals were used to assess both navitoclax pharmacokinetics (*n* = 14) and potential navitoclax-induced thrombocytopenia (*n* = 17). On the other hand, 26 animals underwent tMCAO and were randomized to receive either vehicle or navitoclax, but 6 were excluded from the study because they met one of the exclusion criteria: (1) no ischemia (CP reduction < 50% from baseline, *n* = 3); (2) no reperfusion (CP did not reach the baseline after filament withdrawal, *n* = 0); (3) death before the time limit established (*n* = 2); and (4) no infarction in spite of a right ischemia–reperfusion pattern (*n* = 1). Therefore, 10 animals receiving vehicle (60% phosal 50 PG, 30% polyethylene glycol 400, and 10% ethanol) and 10 animals receiving navitoclax (6 × 10 mg/kg) via i.p. injections (1 mL) every other day between days 3 and 13 after tMCAO were included.

### 4.3. Navitoclax Pharmacokinetics and Hemogram

Navitoclax (10 or 30 mg/kg) was injected (i.p.) and tail vein blood samples were taken at 0.5, 1, 2, 4, 8, 12, 24, 36, and 48 h. Animals were euthanized at 24 h or 48 h by intracardiac injection of KCl (200 mg/kg) to obtain the brain. Serum and brain navitoclax levels were determined using liquid chromatography with tandem mass spectrometry (UPLC-MS/MS 6460 System, Agilent Technologies, Santa Clara, CA, USA) [29]. On the other hand, blood samples were obtained before and after (24 h and 48 h) navitoclax injection to obtain the hemogram (DxH 900 Haematology Analyzer, Beckman Coulter, Brea, CA, USA).

### 4.4. Ischemic Stroke: Transient Focal Cerebral Ischemia

The animal model (tMCAO) was always carried out from 9:00 h to 14:00 h, that is, during the resting phase of the circadian cycle in rats. Animals were anesthetized by intraperitoneal (i.p.) injection of 5 mg/kg diazepam and 100 mg/kg ketamine. Inhalatory anesthesia was maintained with 0.5–1% sevoflurane in 80% medicinal air plus 20% O_2_. Right tMCAO was performed by following the intraluminal nylon filament procedure as originally described [30] and adapted to our experimental setup [31]. A silicone rubber-coated monofilament (403956 or 404156; Doccol Corporation, Sharon, MA, USA) was used. The procedure included continuous monitoring of cerebrocortical laser-Doppler flow (CP), and core temperature. For mechanical recanalization, the monofilament was withdrawn after 60 min of MCAO and reperfusion was monitored for 10 min. Buprenorphine (s.c., 0.05 mg/kg) was used to provide analgesia. The animals were subjected to neurofunctional evaluation at baseline and 24 h, 7 days, and 14 days after the ischemic insult. Tail vein blood samples were obtained at baseline and on days 4, 7, and 14. Then, the animals were euthanized by intracardiac injection of KCl (200 mg/kg) to obtain the brain and post-process it according to specific requirements for each determination.

### 4.5. Brain Damage Assessment: Neurofunctional Performance and Infarct Size

Neurological function was assessed by means of neurofunctional score and tape removal tests. The animals were subjected to a neurological evaluation that consisted of testing sensitive, motor, and reflex skills as described previously [32]. Scores range from zero to 12, zero indicating no impairment and 12 indicating extreme impairment in neurological function. The tape removal test (also known as bilateral asymmetry test) is a test of tactile extinction probing sensory neglect [33]. Two tape strips of equal size (12 mm long and 8 mm wide) were applied with equal pressure to the forepaws. The times for reaction and for the removal of the right (healthy) and left (affected) forepaws were measured, as well as the total time spent on tape removal.

Fresh frozen cryostat-cut brain coronal sections 0.2 to −1.8 mm from the bregma were obtained (20 µm thick; CM 1950 cryostat, Leica Biosystems, Nussloch, Germany), stained with 0.5% thionine as previously reported [34], and observed at 2X magnification in a DMD108 digital microscope (Leica Biosystems, Wetzlar, Germany). An investigator blinded to the experimental groups analyzed the captured images using ImageJ NIH software, version 1.53t, to measure the infarct area based on the glial scar [34].

### 4.6. Brain Cell Senescence Assessment: Senescence-Associated β-Galactosidase and Lipofuscin Staining

SA-β-gal staining was carried out in brain coronal sections by using the Senescence β-Galactosidase Staining Kit (#9860, Cell Signaling, Danvers, MA, USA) according to the manufacturer’s instructions. Briefly, sections were fixed and then incubated with the β-Galactosidase Staining Solution (pH = 6; overnight at 37 °C). Afterward, sections were counterstained for 10 min with 0.1% Nuclear Fast Red (NFR, Sigma-Aldrich, Darmstadt, Germany).

Lipofuscin staining was carried out using the Sudan Black B (SBB, Sigma-Aldrich, Darmstadt, Germany) method. Briefly, brain coronal sections were prefixed (4% paraformaldehyde) washed with PBS, and incubated in increasing ethanol concentrations (EtOH, 50%, 60%, and 70%). Freshly prepared SBB (0.7 g in 100 mL 70% EtOH and double filtered) was dropped on a clean slide. The slide with the specimen was placed facing down on the SBB drop and allowed to react for 8 min. The tissue was then embedded into 50% EtOH, transferred and counterstained with 0.1% NFR for 10 min. Slides were mounted into 40% glycerol.

ImageJ NIH software version 1.53t was used for quantification by an investigator blinded to the experimental groups and selected regions, cortex and caudate putamen.

### 4.7. RT-qPCR Analysis of Gene Expression

A 2 mm thick brain coronal section (0.2 to −1.8 mm from bregma) was obtained and both ischemic and non-ischemic cortex and caudate putamen samples were dissected, flash-frozen in liquid N_2_, lyophilized, and ground to obtain brain powder. Total RNA was isolated using the TRIZOL reagent according to the manufacturer’s instructions (Sigma-Aldrich). The cDNA used as template for amplification in the qPCR assay was obtained by the reverse transcription reaction using PrimeScriptTM RT reagent Kit (Takara Bio, Kusatsu, Japan) according to the manufacturer’s protocol. The gene expression was analyzed using TB Green^®^ Premix Ex TaqTM or Premix Ex TaqTM kits for primers or taqman probes, respectively (Takara Bio), in a ViiA 7 Real-Time PCR System (Thermo Fisher Scientific, Waltham, MA, USA). Each reaction was run in triplicate, the threshold cycle was determined, and the relative gene expression was calculated with the Schmittgen and Livak comparative method [35], using Actb as a reference gene. The specific primers and TaqMan probes used are shown in Table 2.

### 4.8. Statistical Analysis

Data are expressed as mean ± standard error of the mean (SEM), except for neurofunctional score, expressed as median (Q1, Q3). Data analysis was performed using GraphPad Prism 10 software (GraphPad Software, Boston, MA, USA). Differences were considered significant at *p* < 0.05.

## Figures and Tables

**Table 1:** Physiological parameters in the two experimental groups of rats subjected to transient middle cerebral artery occlusion, before i.p. treatment with navitoclax or vehicle.

	Vehicle	Navitoclax (6 × 10 mg/kg)
**CP (%)** ^a^		
Baseline	100	100
Ischemia	41.6 ± 8.4	38.8 ± 5.4
Reperfusion	138.9 ± 25.4	128.3 ± 13.8
Glycemia (mg/dL)	118.3 ± 2.8	122.0 ± 3.6
Weight (g)	343.9 ± 5.8	340.9 ± 6.6

Data are expressed as mean ± SEM from 8 to 10 rats. ^a^ CP, cortical perfusion.

**Table 2:** Oligonucleotides used for qRT-PCR.

Target Gene	TaqMan Probe	Forward Primer Sequence (5′-3′)	Reverse Primer Sequence (5′-3′)
*Chk1*		CATGTTTCCAGTTGGCCTCT	TCTTCTTGTCTGGGCGACTT
*Chk2*		CTTTCGCATCTTCAGGGAAA	AGTGAAAGTGCGATTTCAGAGTT
*Actb*		TTCAACACCCCAGCCATGT	GTGGTACGACCAGAGGCATACA
*Cdkn2a/p16*	Rn00580664_m1		
*Cdkn1a/p21*	Rn00589996_m1		
*Il1b*	Rn00580432_m1		
*Tnfa*	Rn01525859_g1		
*Actb*	Rn00667869_m1		

## Data Availability

The data presented in this study are available on request from the corresponding author. The data are not publicly available due to [privacy and confidentiality restrictions].

## Supplementary Materials

The following supporting information can be downloaded at https://www.mdpi.com/article/10.3390/ph19030431/s1. Figure S1. Effects of navitoclax on neurofunctional impairment in stroke adult male Wistar rats 24 h, 7 and 14 days after transient middle cerebral artery occlusion (tMCAO). Figure S2. Effects of navitoclax on brain infarct size in adult male Wistar rats 14 days after tMCAO. Figure S3. Effects of navitoclax on lipofuscin accumulation in the brain of adult male Wistar rats 14 days after transient middle cerebral artery occlusion (tMCAO). Figure S4. Effects of navitoclax on the mRNA expression of cyclin-dependent kinase inhibitors (p16 and p21) in the brain of adult male Wistar rats 14 days after transient middle cerebral artery occlusion (tMCAO). Figure S5. Effects of navitoclax on the mRNA expression of SASP cytokines (IL-1β and TNF-α) in the brain of adult male Wistar rats 14 days after transient middle cerebral artery occlusion (tMCAO).

## Abbreviations

The following abbreviations are used in this manuscript:

BBB	blood–brain barrier
Chk1	Checkpoint kinase 1
Chk2	Checkpoint kinase 2
CNS	central nervous system
CP	cortical perfusion
NFR	Nuclear Fast Red
SA-β-gal	senescence-associated β-galactosidase
SASP	senescence-associated secretory phenotype
SBB	Sudan black b
SCAPs	senescent cell anti-apoptotic pathways
SIPS	stress-induced premature senescence
tMCAO	transient middle cerebral artery occlusion
