# Supplementary material for: The Senolytic Drug Navitoclax Protects the Brain After Experimental Ischemic Stroke
Source: Pharmaceuticals (Basel). 2026 Mar 6;19(3):431. doi: 10.3390/ph19030431 (PMC13028689; doi:10.3390/ph19030431)
Supplement: Supplementary file 1 [file pharmaceuticals-19-00431-s001.zip › pharmaceuticals-4155809-supplementary.pdf]

The Senolytic Drug Navitoclax Protects the Brain After Experimental Ischemic Stroke

Dianoush Falahatgaroshibi [1,2,†], Júlia Baixauli-Martín [1,†,‡], María C. Burguete [1,3], Mikahela A. López-Morales [1,4,*], Alicia Aliena-Valero [1], José E. Peris [5], Juan B. Salom [1,3]

[1] Unidad Mixta de Investigación Cerebrovascular, Instituto de Investigación Sanitaria La Fe, 46026 Valencia, Spain
[2] Departamento de Biotecnología, Universidad Politécnica de Valencia, 46022 Valencia, Spain
[3] Departamento de Fisiología, Universidad de Valencia, 46100 Burjassot, Spain
[4] Departamento de Fisioterapia, Universidad de Valencia, 46010 Valencia, Spain
[5] Departamento de Farmacia y Tecnología Farmacéutica y Parasitología, Universidad de Valencia, 46100 Burjassot, Spain
† These authors contributed equally to this work.
‡ Current address: Unidad de Investigación, Hospital Universitario Santa Cristina, Instituto de Investigación Sanitaria Princesa, 28009 Madrid, Spain

* Correspondence: mikahela_lopez@iislafe.es

Supplementary material

Supplementary Figures S1, S2, S3, S4, and S5.

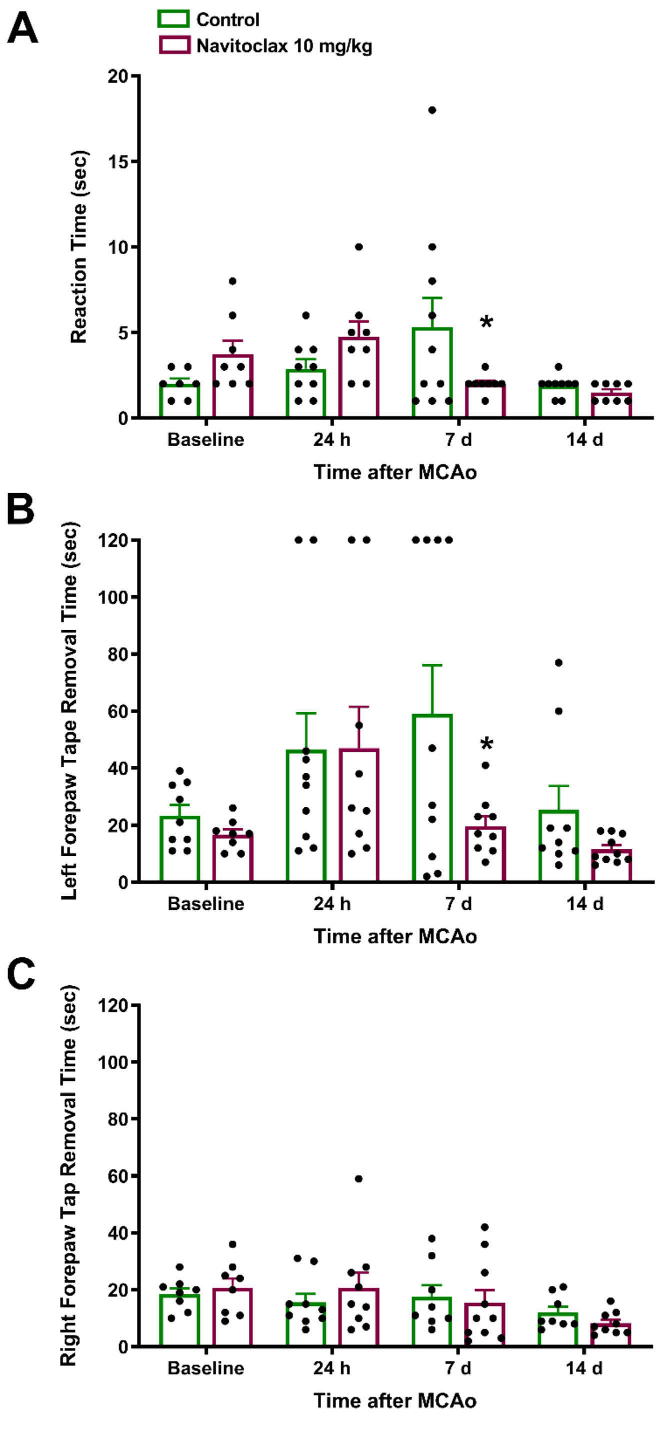

Figure S1. Effects of navitoclax on neurofunctional impairment in stroke adult male Wistar rats 24 h, 7 and 14 days after transient middle cerebral artery occlusion (tMCAO). The animals were injected i.p. with navitoclax (10 mg/kg) or vehicle every other day between days 3 and 13 after tMCAO. **(A)** Reaction time, **(B)** left forepaw tape removal time, and **(C)** right forepaw tape removal time in the bilateral asymmetry test. Mixed-effects model followed by Šidák test. *$p < 0.05$, significantly different from vehicle. Data are expressed as mean ± SEM.

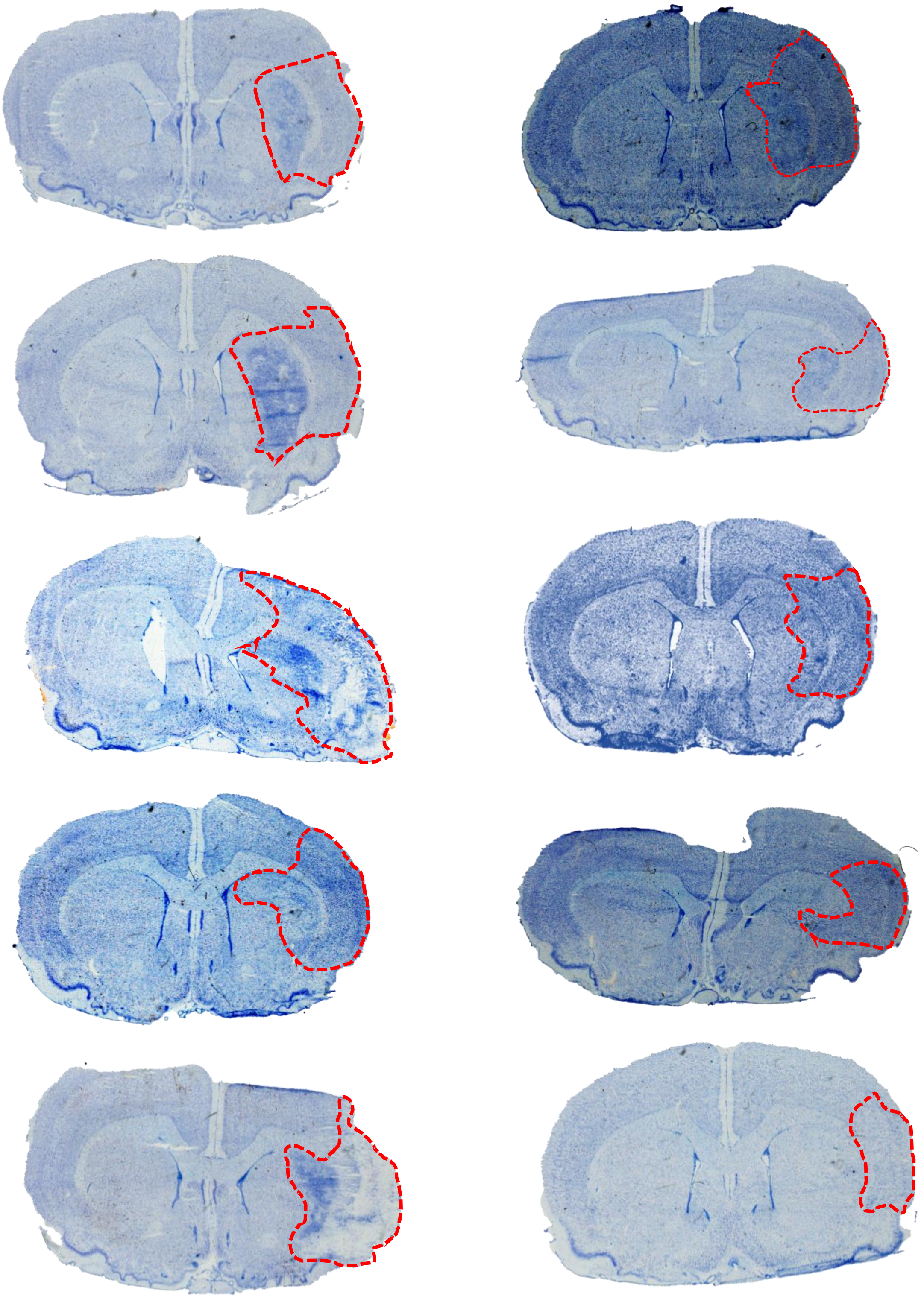

Figure S2. Effects of navitoclax on brain infarct size in adult male Wistar rats 14 days after tMCAO. The animals were injected i.p. with navitoclax (10 mg/kg) or vehicle every other day between days 3 and 13 after tMCAO. Representative macroscopic images of thionine-stained brain coronal sections (0.2 to–1.8 mm from the bregma) in five vehicle-treated (left) and five navitoclax-treated (right) animals. The infarct areas are outlined by red dotted lines.

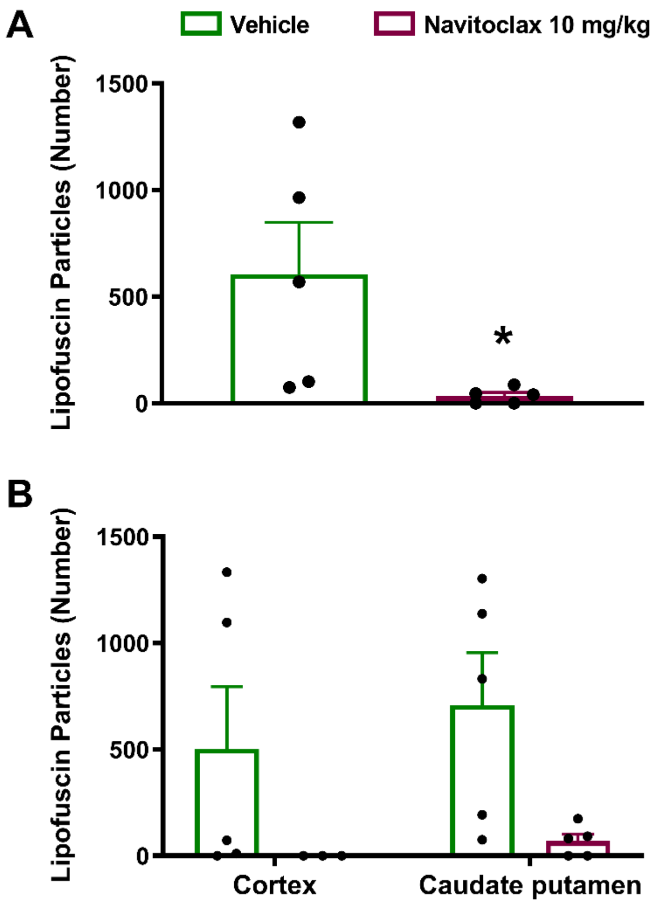

Figure S3. Effects of navitoclax on lipofuscin accumulation in the brain of adult male Wistar rats 14 days after transient middle cerebral artery occlusion (tMCAO). The animals were injected i.p. with navitoclax (10 mg/kg) or vehicle every other day between days 3 and 13 after tMCAO. **(A)** Lipofuscin particles in coronal sections (0.2 to−1.8 mm from the bregma) of vehicle- and navitoclax-treated animals. Two-tailed Student's t-test. *$p < 0.05$, significantly different from vehicle. **(B)** Regional quantification of lipofuscin particles in the cortex and caudate putamen. Two-way ANOVA followed by Tukey's test. Data are expressed as mean ± SEM.

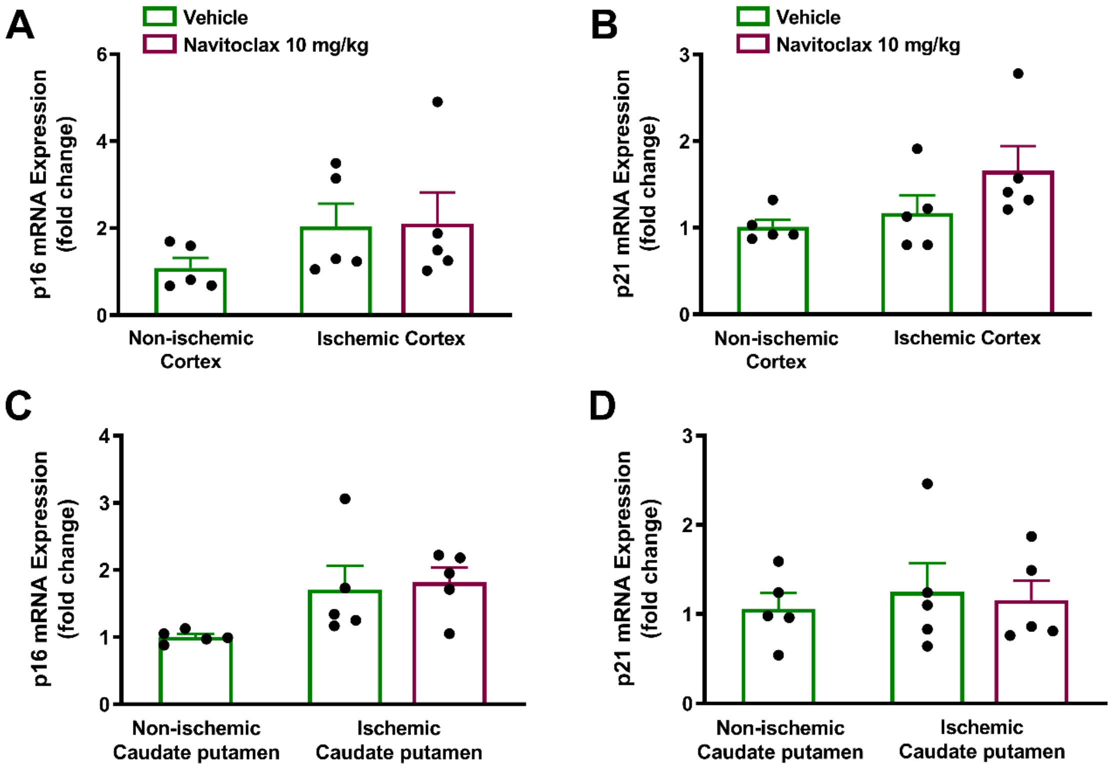

Figure S4. Effects of navitoclax on the mRNA expression of cyclin-dependent kinase inhibitors (p16 and p21) in the brain of adult male Wistar rats 14 days after transient middle cerebral artery occlusion (tMCAO). The animals were injected i.p. with navitoclax (10 mg/kg) or vehicle every other day between days 3 and 13 after tMCAO. **(A)** p16 expression in non-ischemic and ischemic cortex of vehicle- and navitoclax-treated animals. **(B)** p21 expression in non-ischemic and ischemic cortex of vehicle- and navitoclax-treated animals. **(C)** p16 expression in non-ischemic and ischemic caudate putamen of vehicle- and navitoclax-treated animals. **(D)** p21 expression in non-ischemic and ischemic caudate putamen of vehicle- and navitoclax-treated animals. One-way ANOVA followed by Tukey’s test. Data are expressed as mean ± SEM.

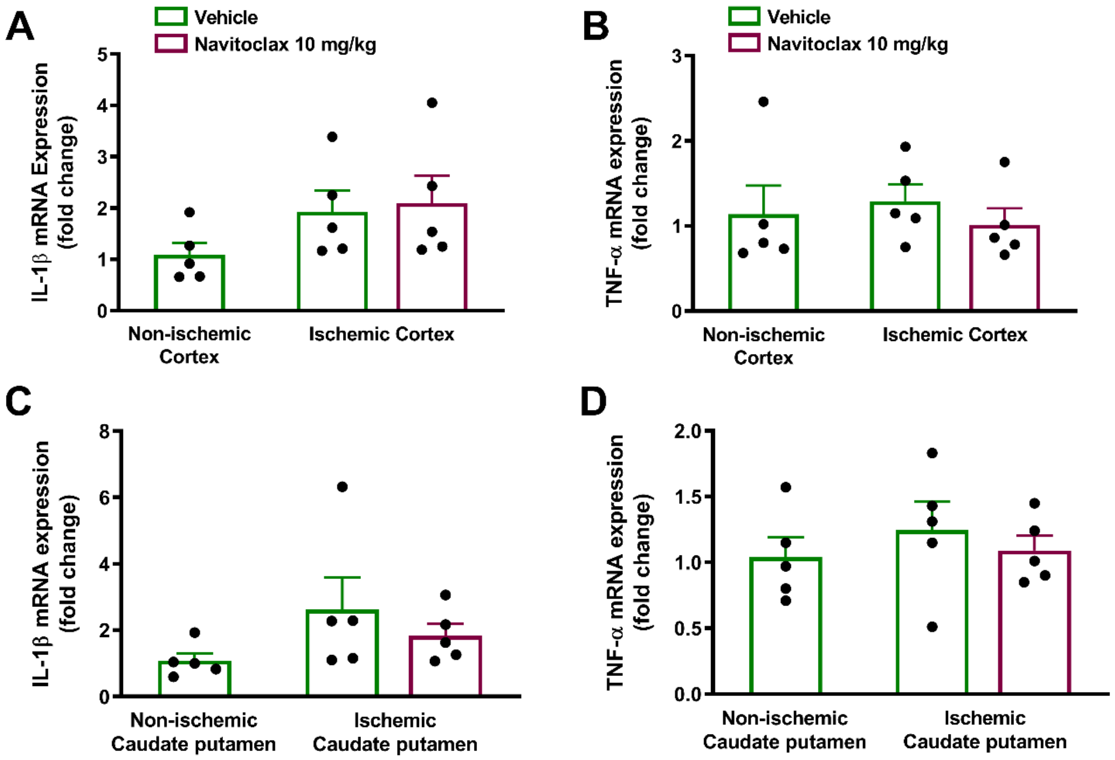

Figure S5. Effects of navitoclax on the mRNA expression of SASP cytokines (IL-1β and TNF-α) in the brain of adult male Wistar rats 14 days after transient middle cerebral artery occlusion (tMCAO). The animals were injected i.p. with navitoclax (10 mg/kg) or vehicle every other day between days 3 and 13 after tMCAO. **(A)** IL-1β expression in non-ischemic and ischemic cortex of vehicle- and navitoclax-treated animals. **(B)** TNF-α expression in non-ischemic and ischemic cortex of vehicle- and navitoclax-treated animals. **(C)** IL-1β expression in non-ischemic and ischemic caudate putamen of vehicle- and navitoclax-treated animals. **(D)** TNF-α expression in non-ischemic and ischemic caudate putamen of vehicle- and navitoclax-treated animals. One-way ANOVA followed by Tukey's test. Data are expressed as mean ± SEM.
